# Melanuria

**Published:** 1910-10-29

**Authors:** 


### MELANURIA.

There are not a few conditions in which darkening of the urine may occur either spontaneously on standing or by means of the addition of reagents; of these the best known perhaps are carboluria, alkaptonuria, and melanuria. There is little likelihood of these three conditions being confused with one another, for alkaptonuria dates from birth and has attention drawn to it by the fact that the child's diapers become blackened; carboluria will not occur unless the patient is receiving carbolic acid from an external source, either by the mouth or by means of a lotion or a dressing, so that when in an adult the urine is found to darken spontaneously without obvious cause it is most likely to be due to melanuria. To confirm this suspicion the specimen should have a few drops of moderately concentrated solution of ferric chloride added to it when, if melanuria is present, there will be blackening which does not occur with the other conditions. This blackening with ferric chloride is a very delicate test, and it is almost the only one which is really trustworthy. If melanuria exists it is practically certain that the patient is suffering from a melanotic sarcoma, and the value of the recognition of the urinary reaction may therefore be considerable in doubtful cases, though it should always be borne in mind that it is by no means every case of melanotic sarcoma that produces melanuria. The presence of melanuria indicates melanotic sarcoma, but its absence does not exclude such sarcoma.

Langdon Brown has recently recorded two cases in which the value of the test was obvious. The first was a woman aged fifty-three, who for three months had been suffering from pain in her left side. The percussion note over the left lung became impaired with loss of tactile vocal fremitus, and loss of breath and voice sounds. No rub was heard. The abnormal signs slowly increased, the pain persisted, and the patient began to be very short of breath. There had been no pyrexia throughout. Notwithstanding signs which might otherwise have suggested a large pleural effusion, there was hardly any displacement of the heart. This suggested new growth within the thorax. The ferric chloride test applied to the urine gave a decided black reaction; on searching for evidence of a primary focus, a black mole, the size of a florin, was found on the right side of the abdomen, the patient said it had been there as long as she could remember, but she thought it had grown larger lately. On exploring the chest with a trocar and cannula only about two ounces of blackish fluid were obtained, and on examining this microscopically large cells containing distinct grains of melanotic pigment were found, and the diagnosis of melanotic sarcoma was confirmed. Death occurred about a month later.

The other patient was a man aged fifty-seven, in whom the urine also gave the black ferric chloride reaction. He stated he had been getting thinner for weeks, and had been suffering from shortness of breath. Twelve months previously he had been in a hospital, where five pints of blood-stained fluid were removed from the left side of his chest. Three years previous to this the right eye had been excised, though for what reason was not known for certain at the time. The left side of the thorax was now bulged, and exhibited deficient respiratory movements. There were enlarged veins over the upper part of the chest and abdomen, the blood current being the reverse of normal. Several small subcutaneous nodules could be felt on the chest and abdomen, and there was also one on the left clavicle. On percussion there was dulness from the second rib out to the axillary fold on the left side, reaching two finger-breadths to the right of the sternum, and also to one inch above the angle of the left scapula behind. The cardiac impulse was felt in the fourth left space close to the sternum. Over the dull area the tactile vocal fremitus was greatly diminished, the voice sounds were muffled, and the breath sounds inaudible. The physical signs were decidedly suggestive of intra-thoracic new growth; the fact that the eye had been removed some years previously suggested melanotic sarcoma and the melanin reaction in the urine confirmed this. If any doubt had remained it was removed by the excision of one of the subcutaneous nodules which exhibited the characters of melanotic sarcoma. The patient grew speedily worse, and death occurred seven weeks later. At the post-mortem examination an enormous mass of melanotic sarcoma was found filling the left side of the chest, replacing the greater part of the left lung tissue and extending across the middle line to the right and perforating the diaphragm downwards. The mediastinal, and lower cervical glands, the ribs, and the xiphoid cartilage were involved in the new growth, and there were nodules in the mesenteric and retro-peritoneal glands, the mucous membrane of the small intestine, the bladder, and the pancreas.

